# Rapamycin Reverses the Hepatic Response to Diet‐Induced Metabolic Stress That Is Amplified by Aging

**DOI:** 10.1111/acel.70395

**Published:** 2026-02-07

**Authors:** Aaron Havas, Adarsh Rajesh, Xue Lei, Jessica Proulx, Karl N. Miller, Adam Field, Andrew Davis, Marcos Garcia Teneche, Armin Gandhi, Jin Lee, Gen‐Sheng Feng, Peter D. Adams

**Affiliations:** ^1^ Sanford Burnham Prebys Medical Discovery Institute La Jolla California USA; ^2^ University of California San Diego La Jolla California USA

## Abstract

Aging is associated with increased susceptibility to metabolic stress and chronic liver disease, yet the interactions between age and metabolic stressors and the potential for ameliorating interventions remain incompletely understood. Here, we examined the hepatic response of young (7‐month‐old) and old (25‐month‐old) C57BL/6 male mice to a 9‐week high‐fat diet (HFD) and assessed whether rapamycin, a well‐established pro‐longevity intervention, could mitigate age‐exacerbated effects. While both age groups developed metabolic‐associated steatohepatitis (MASH), older mice displayed more severe hepatic steatosis, inflammation, and transcriptional dysregulation. Transcriptomic profiling of whole livers and purified hepatocytes revealed that aging amplifies HFD‐induced inflammatory and metabolic gene expression changes, including activation of immune pathways and suppression of metabolic pathways. Notably, treatment of aging mice with rapamycin reversed the majority of HFD‐driven transcriptional alterations, including upregulation of pro‐inflammatory regulators such as Stat1, and dysregulation of metabolic gene networks. Rapamycin also reduced hepatosteatosis, total body weight, and a tumorigenic transcriptomic signature associated with hepatocellular carcinoma risk. These findings demonstrate that aging intensifies hepatic sensitivity to dietary metabolic stress and identify rapamycin as a promising therapeutic to counteract age‐related liver dysfunction and metabolic dysfunction‐associated steatotic liver disease (MASLD) progression.

## Graphical abstract

Aging exacerbates high‐fat diet (HFD) induced hepatic steatosis, and transcriptional dysregulation of pathways including inflammation, immune, and metabolic regulation in mouse liver and hepatocytes. The addition of rapamycin to HFD reversed most HFD‐driven gene‐expression changes in old mice, including those linked to metabolic dysfunction and inflammation, as well as hepatosteatosis and body weight gain in old mice.

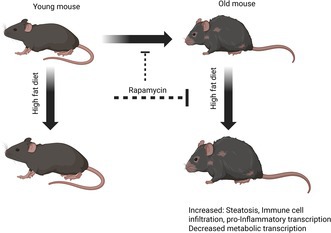

## 1 Introduction

Metabolic dysfunction associated steatotic liver disease (MASLD) is a progressive disease, including liver steatosis and inflammatory metabolic dysfunction associated steatohepatitis (MASH). MASLD is a growing public health burden through its association with cirrhosis and liver cancer development (Stefan et al. 2025; Targher et al. 2025). Obesity and associated metabolic dysfunction fueled by poor diet (e.g., high‐fat diet (HFD), ultraprocessed foods) and sedentary lifestyles have become epidemic across all age groups. Obesity increases the risk of MASLD.

As the liver ages, it exhibits structural and functional changes including reduced volume, diminished blood flow, and mild fibrosis (Harada et al. 2021; Schmucker 2005). Immune function becomes dysregulated, with increased immune cell infiltration. Aged liver also exhibits altered nutrient and insulin sensing, mitochondrial dysfunction, diminished regenerative capacity, and disrupted metabolic homeostasis (Le Couteur et al. 2025; Singh et al. 2008; Guo et al. 2022; Lin et al. 2023). Older individuals demonstrate altered fat distribution, increased susceptibility to severe steatosis, and greater risk of MASLD progression and hepatocellular carcinoma (Liang et al. 2024; Younossi et al. 2023; Shah et al. 2023). Together, these changes reduce the liver's resilience to injury and disease, underscoring its vulnerability in aging (Maeso‐Diaz et al. 2022).

While obesity‐ and metabolic dysfunction‐related pathologies have been extensively modeled, most studies examine aging and metabolic stress in isolation, failing to capture their intersection. There remains a critical need to understand how aging modifies the transcriptional and physiological landscape under conditions of dietary metabolic stress, and to identify interventions capable of mitigating these compounded effects.

Rapamycin, a selective inhibitor of mTORC1, has emerged as one of the most promising pharmacologic candidates for promoting healthy aging and extending lifespan. Across a wide range of model organisms, rapamycin has been shown to robustly increase longevity, even when administered later in life (Harrison et al. 2009; Sharp and Strong 2023; Schinaman et al. 2019). Its pro‐longevity effects are attributed to its ability to modulate key aging‐related processes, including altered growth signaling, enhanced autophagy, suppression of chronic inflammation, and improved mitochondrial and metabolic function (Zhang et al. 2021). In some metabolic disease models, rapamycin reduces adiposity and protects against hepatic steatosis and dyslipidemia (Zhao et al. 2025; Chang et al. 2009; Bitto et al. 2021; Leontieva et al. 2014a, 2014b). These effects indicate a potent role in mitigating the consequences of metabolic stress. However, few studies have investigated the impact of rapamycin on diet‐induced metabolic stress in aged mice. Although Leontieva and Blagosklonny showed that rapamycin is of benefit in old mice on HFD (Leontieva et al. 2014a, 2014b), reflected in decreased weight gain and/or extended lifespan, the molecular insights were limited.

In this study, we interrogated how aging modifies hepatic responses to metabolic stress in the form of HFD, and whether rapamycin can attenuate these effects. Using a comparative model of young and old mice subjected to HFD, we show that older mice displayed more severe HFD‐induced hepatic steatosis, inflammation, and transcriptional dysregulation. Rapamycin treatment reversed these deleterious HFD‐induced transcriptional changes, reduced body and liver mass, and improved hepatic histopathology in aged mice. These findings suggest that rapamycin can counteract the exaggerated transcriptional and physiological consequences of metabolic stress in aging, expanding our understanding of its therapeutic potential for age‐related metabolic disease.

## 2 Results

**FIGURE 1:**
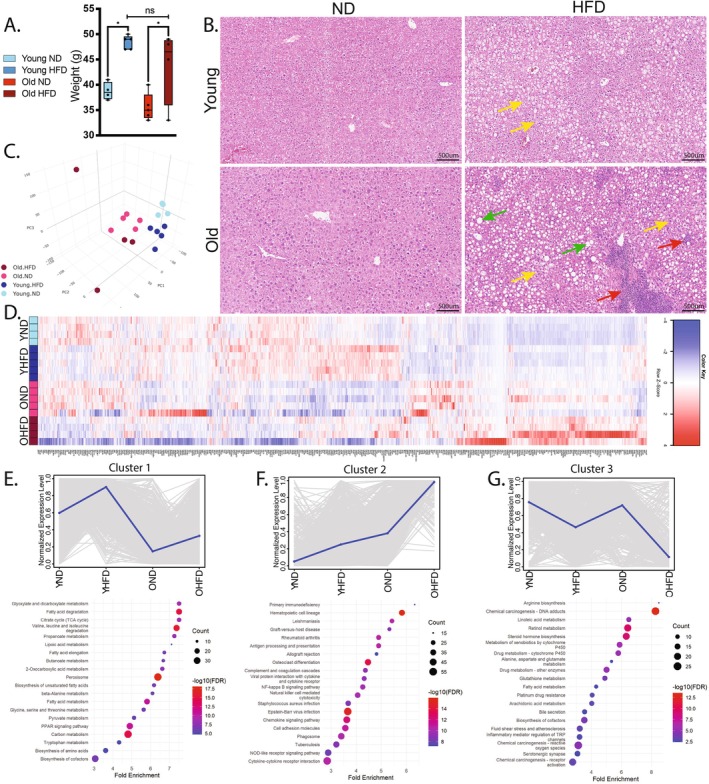
Aging exacerbates high‐fat diet (HFD)‐induced metabolic stress responses. (A) Body weight distribution (grams) of young (5‐month‐old) and aged (22‐month‐old) mice after 9 weeks of HFD or control diet. (B) Representative H&E‐stained liver sections. Green arrows indicate fat accumulation; yellow arrows indicate hepatocyte ballooning and red arrows indicate immune infiltration. (C) Principal component analysis (PCA) of whole‐liver RNA‐seq data. (D) Heatmap showing all differentially expressed genes (DEGs). (E–G) Gene clustering analysis (top panels) with associated enriched KEGG pathways (bottom panels). Statistical analysis of body weights was performed using one‐way ANOVA with post hoc Tukey's test; *p* < 0.05 is indicated by an asterisk (*).

To investigate how aging impacts the response to dietary metabolic stress, we examined the effects of a HFD on young (5‐month‐old) and old (22‐month‐old) C57BL/6 male mice over a 9‐week feeding period. Both age groups exhibited comparable weight gain on HFD, with no significant differences in body weight between age groups at collection (Figure 1A). However, histological analysis revealed notable age‐dependent differences: while both young and old mice on HFD developed signs of hepatic steatosis and MASH, as evidenced by fat accumulation (Figure 1B, green arrows), hepatocyte ballooning (Figure 1B, yellow arrows), and immune infiltration (Figure 1B, red arrows, Figure S1), these features were more pronounced in the older cohort (Singh et al. 2008; Zhao et al. 2024; Nunes‐Souza et al. 2016; Kim et al. 2016; Fontana et al. 2013; Sheedfar et al. 2014).

To investigate how aging influences the hepatic response to HFD at the molecular level, we performed RNA sequencing (RNA‐seq) on whole liver samples. Principal component analysis (PCA) revealed distinct clustering of all groups, albeit with the highest variability observed in the old HFD cohort, suggesting an age‐and diet‐dependent divergence in transcriptome (Figure 1C). Differential gene expression analysis identified over 4000 differentially expressed genes (DEGs) across any cohort, with the most substantial difference—3954 DEGs—between young ND and old HFD groups (Figure 1D). Clustering of DEGs revealed three major expression trajectories. Cluster 1, encompassing 1190 transcripts upregulated by HFD in both age groups, was enriched for KEGG metabolic pathways, including fatty acid degradation and peroxisome and carbon metabolism (Figure 1E). Cluster 2, comprising 1244 transcripts elevated with age and further amplified by HFD, was enriched for inflammatory and immune activation pathways such as graft‐versus‐host disease and infection responses (Figure 1F). Cluster 3, containing 627 transcripts downregulated by HFD in both age groups, included genes involved in metabolism, cytochrome P450, and steroid hormone biosynthesis (Figure 1G). These data suggest that while the transcriptional response to HFD shares core similarities between young and old livers, aging amplifies some inflammatory and metabolic changes as identified in clusters 2 and 3, underscoring the heightened vulnerability of aged livers to metabolic stress, also observed at the histological level.

**FIGURE 2:**
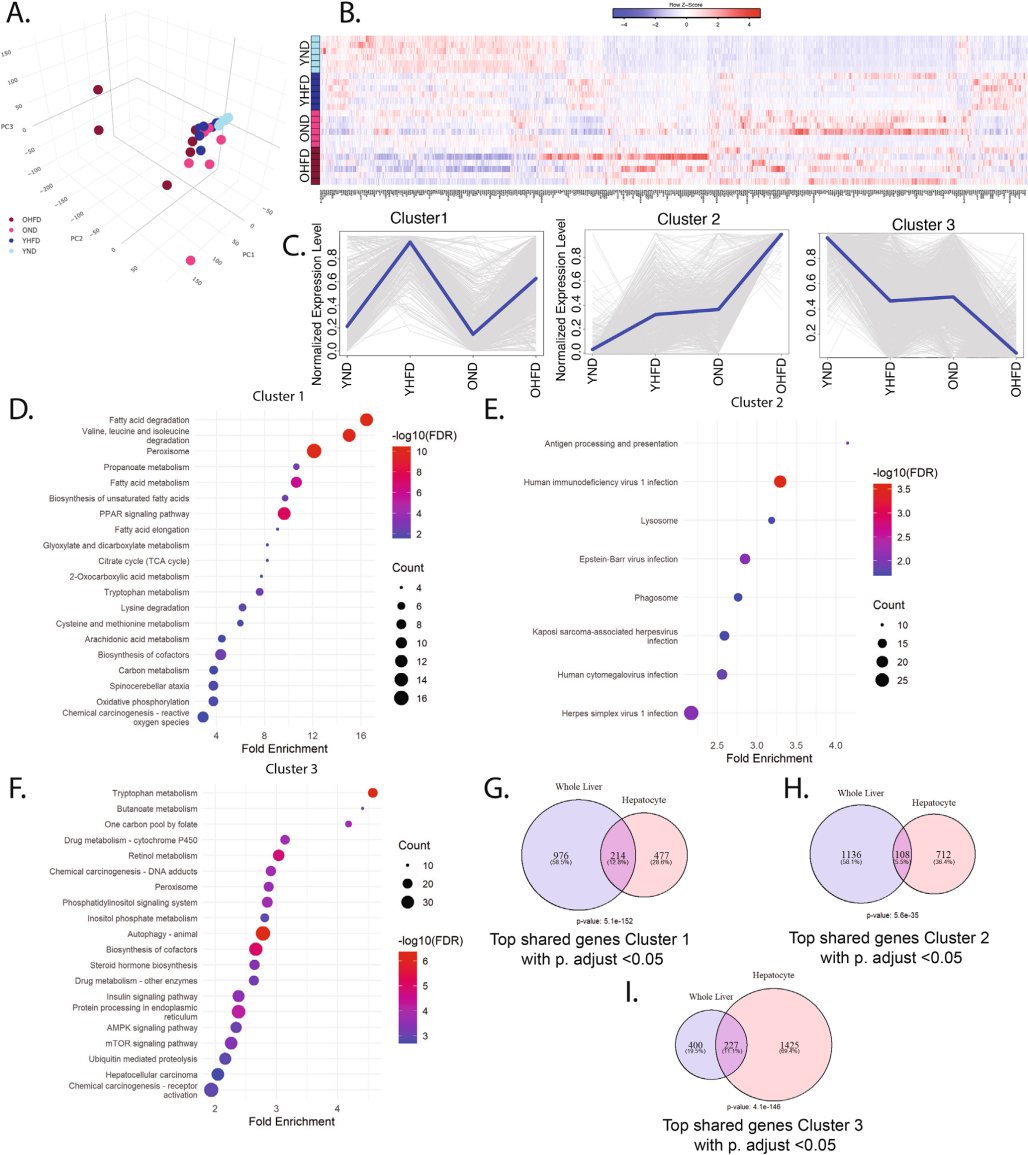
Aged hepatocytes exhibit hyperbolic metabolic dysfunction and heightened proinflammatory signaling in response to HFD. Hepatocytes were isolated from 5‐month‐old (young) and 22‐month‐old (aged) mice following 9 weeks of control or high‐fat diet (HFD) treatment. (A) Principal component analysis (PCA) of hepatocyte RNA‐seq data. (B) Heatmap of all differentially expressed genes (DEGs). (C) Top three gene expression clusters, showing patterns based on age and diet. (D–F) Enriched KEGG pathways corresponding to clusters shown in (C). (G–I) Venn diagrams comparing genes from whole liver and isolated hepatocyte datasets within analogous clusters 1–3, respectively.

Hepatocytes are the major drivers of liver metabolic functions and exhibit ballooning and fat accumulation in response to HFD (Figure 1B yellow and green arrows). However, age and diet induced increased immune‐cell population within the liver (Figure 1B red arrow, Figure S1) and this was confirmed by CYBRSORTx (Newman et al. 2015) analysis, which indicated a number of transcriptomic changes, including a marked increase in CD8+ T signature in old livers and resting dendritic cells in old HFD livers (Figure S2). Therefore, to mitigate the potential impact of the immune cell population and to achieve a hepatocyte‐specific understanding of transcriptional responses to HFD, we performed RNA‐seq on isolated hepatocytes from an additional cohort of mice subjected to the same experimental treatment. PCA again revealed the greatest transcriptional variation and divergence from young normal diet in the old HFD cohort, supported by a heatmap showing 5565 DEGs between any pairwise comparison (Figure 2A,B). Analysis identified three major gene clusters comprising over 50% of the DEGs (Figure 2C). Cluster 1, containing 439 transcripts upregulated by HFD in hepatocytes of both young and old mice, was enriched for metabolic pathways, such as fatty acid degradation, peroxisome signaling, and fat digestion, similar to Cluster 1 from whole‐liver analysis (Figure 2C,D). Cluster 2 included 1033 transcripts elevated with age and further amplified by HFD and was enriched for pathways involved in infection and antigen presentation, again similar to Cluster 2 from whole‐liver analysis (Figure 2C,E). Cluster 3, comprising 1421 transcripts downregulated by HFD in both age groups, was associated with pathways such as metabolism, cytochrome P450, and steroid hormone biosynthesis, echoing the findings from the corresponding whole‐liver Cluster 3 (Figure 2C,F). To better assess the similarity of Clusters 1–3 from whole liver and hepatocytes, we analyzed the overlap of DEGs in the corresponding clusters between whole liver and isolated hepatocytes. While many DEGs are unique to one but not the other, a significant number of genes are shared between whole liver and hepatocytes in all 3 clusters (Figure 2G–I). Overall, these results show that age modifies the liver's response to HFD, in many cases amplifying the effects of HFD (Clusters 2 and 3), and these effects are apparent even in enriched hepatocytes.

**FIGURE 3:**
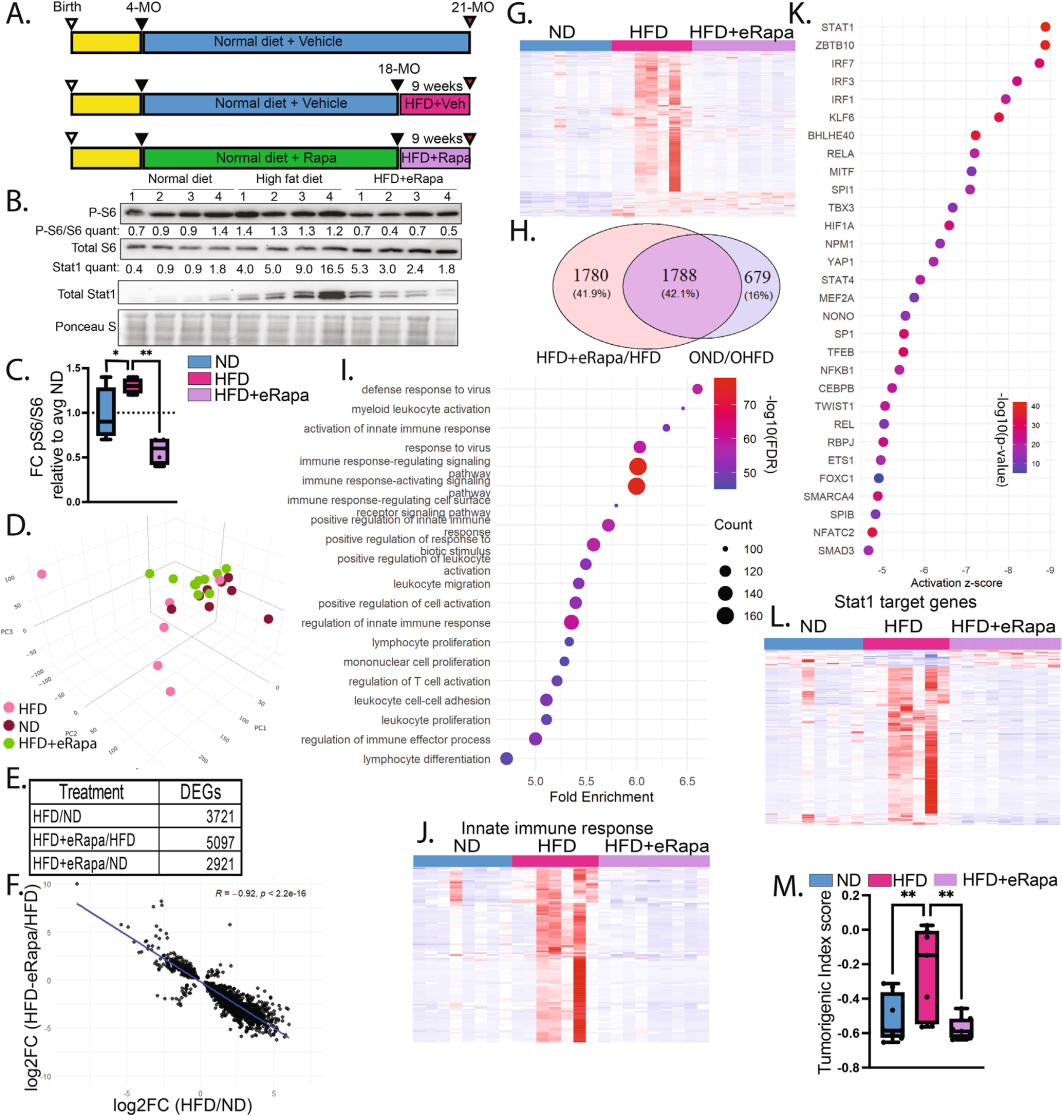
Rapamycin co‐treatment mitigates proinflammatory transcriptional hyperactivation in aged mice exposed to HFD. (A) Protocol: Mice received ND or eRapamycin‐containing diet from 4 to 18 months, followed by 9 weeks on HFD or ND. (B) Western blot of liver tissue. (C) Fold change of p‐S6/S6 compared to control diet. (D) PCA of RNA‐seq from isolated hepatocytes. (E) Number of DEGs across treatment groups. (F) Scatter plot comparing HFD‐induced gene expression changes (HFD + veh vs. ND + veh) to all gene expression changes with eRapamycin (eRapa+HFD vs. HFD + veh). (G) Heatmap of genes upregulated by HFD + vehicle treatment. (H) Venn diagram showing overlap between transcripts elevated by HFD versus ND and those decreased by rapamycin. (I) KEGG enrichment analysis of overlapping genes. (J) Heatmap of innate immune response genes. (K) Top transcription factor pathways predicted by IPA in the overlapping gene set. (L) Heatmap of Stat1 target genes. (M) Tumorigenic index score calculated per mouse. Statistical analyses for p‐S6/S6 ratios and tumorigenic index scores were performed using one‐way ANOVA with post hoc Tukey's test; *p* < 0.05 (*), *p* < 0.1 (**).

Given the interaction between age and HFD, we wondered whether the pro‐longevity intervention rapamycin would mitigate the effects of HFD in aged mice. To address this, we administered rapamycin (42 ppm encapsulated in eudragit, eRapamycin (eRapa)) or vehicle control to C57BL/6 male mice, beginning at 4 months of age to avoid developmental effects. At 18 months, mice were either maintained on ND or switched to HFD ± eRapa for 9 weeks, resulting in three cohorts: ND + vehicle, HFD + vehicle, and HFD + eRapa (Figure 3A). At 21 months, whole livers were collected for histological and tissue analysis, and hepatocytes were isolated for biomarker analysis and RNA sequencing.

Albumin concentration in whole tissue homogenates was assayed by ELISA (normalized to total protein) and showed no significant difference between cohorts (Figure S3A), indicating comparable gross liver function, at least by this measure. Western blot analysis of the ratio of phosphorylated S6 to total S6, a marker of mTOR activity, was significantly reduced in hepatocytes of the HFD‐eRapa group as compared to the HFD + veh cohort, indicating on target activity of rapamycin (Figure 3B,C). PCA of hepatocyte transcriptomes revealed a tendency to distinct clustering of cohorts (Figure 3D). Analysis of mTor target genes showed that HFD + Veh exhibited increased mTor signaling relative to ND + veh, while HFD + eRapa antagonized this trend (Figure S3B–E), further indicating on‐target activity of the eRapa within the diet.

Comparing DEGs, the greatest number (5097) was observed between HFD + eRapa and HFD + veh cohorts (Figure 3D,E). Of note, HFD + eRapa versus ND + veh (2921) showed fewer DEGs than HFD + veh versus ND + veh (3721), consistent with an ability of rapamycin to antagonize the effects of HFD. Indeed, a plot of all genes differentially expressed between HFD + veh versus ND + veh against those genes in HFD + eRapa versus HFD + veh indicated that rapamycin reversed or suppressed most transcriptional changes induced by HFD, with a Pearson correlation of −0.92 between DEGs modulated by HFD + veh and averted by HFD + eRapa (Figure 3F). This is visualized by a heatmap of all genes upregulated in HFD + veh versus ND + veh showing a strong suppression by HFD + eRapa (Figure 3G). One of the main pathways identified to be mainly modulated with age was the metabolism of branch chain amino acids (Valine, Leucine and Isoleucine), which was slightly increased with HFD. This trend remains the same in this experiment yet the addition of eRapa significantly increased this metabolic pathway (Figure S3F–I). Interestingly, genes differentially expressed in young ND versus old ND tended to be regulated in the opposite direction by eRapa on HFD, suggesting eRapa's ability to antagonize age‐associated changes in gene expression even on HFD (Figure S3J).

Previous analysis of whole liver and purified hepatocytes identified Clusters 1 and 2 as upregulated by HFD (Figures 1 and 2). We asked whether these genes are suppressed by rapamycin. Of 2467 genes upregulated by HFD + veh in hepatocytes of old mice, 72% (1788 genes) were downregulated by HFD + eRapa, a highly significant 6.5‐fold enrichment over random (Figure 3H). These genes were enriched for immune‐related processes such as defense response to infection and innate immune activation (Figure 3I), like Cluster 2 induced by age and HFD + veh (Figures 1 and 2). A heatmap of the genes associated with regulation of innate immune response shows the effect of HFD + eRapa to assuage this HFD‐induced activation of this pathway (Figure 3J). Ingenuity Pathway Analysis (IPA) highlighted key pro‐inflammatory transcriptional regulators, including *Stat1*, *Irf3*, and *Rela*, which were downregulated by HFD + eRapa (Figure 3K). A heatmap of *Stat1* target genes exemplifies this repressive effect of HFD + eRapa (Figure 3L). Western blot analysis confirmed elevated *Stat1* protein levels in HFD + veh and a marked reduction in HFD + eRapa (Figure 3B). Assessment of senescence associated secretory phenotype (SASP) genes showed an elevation with HFD + veh that is reduced with HFD + eRapa (Figure S3K,L). To assess the association of these transcriptional changes on risk of cancer, a disease whose incidence is increased with age and metabolic stress, we applied the tumorigenic index (TI) algorithm developed by Wang et al. (2019) which calculates the likelihood of hepatocellular carcinoma (HCC) development based on RNA‐seq data. The TI, elevated by HFD + veh compared to ND + veh, was completely normalized in the HFD + eRapa cohort (Figure 3M). Collectively, these results demonstrate that rapamycin suppresses many of the HFD‐induced pro‐inflammatory and pro‐tumorigenic transcriptional changes in livers of aged mice on HFD, indicating a protective effect against inflammation and associated oncogenic stress in the context of aging.

**FIGURE 4:**
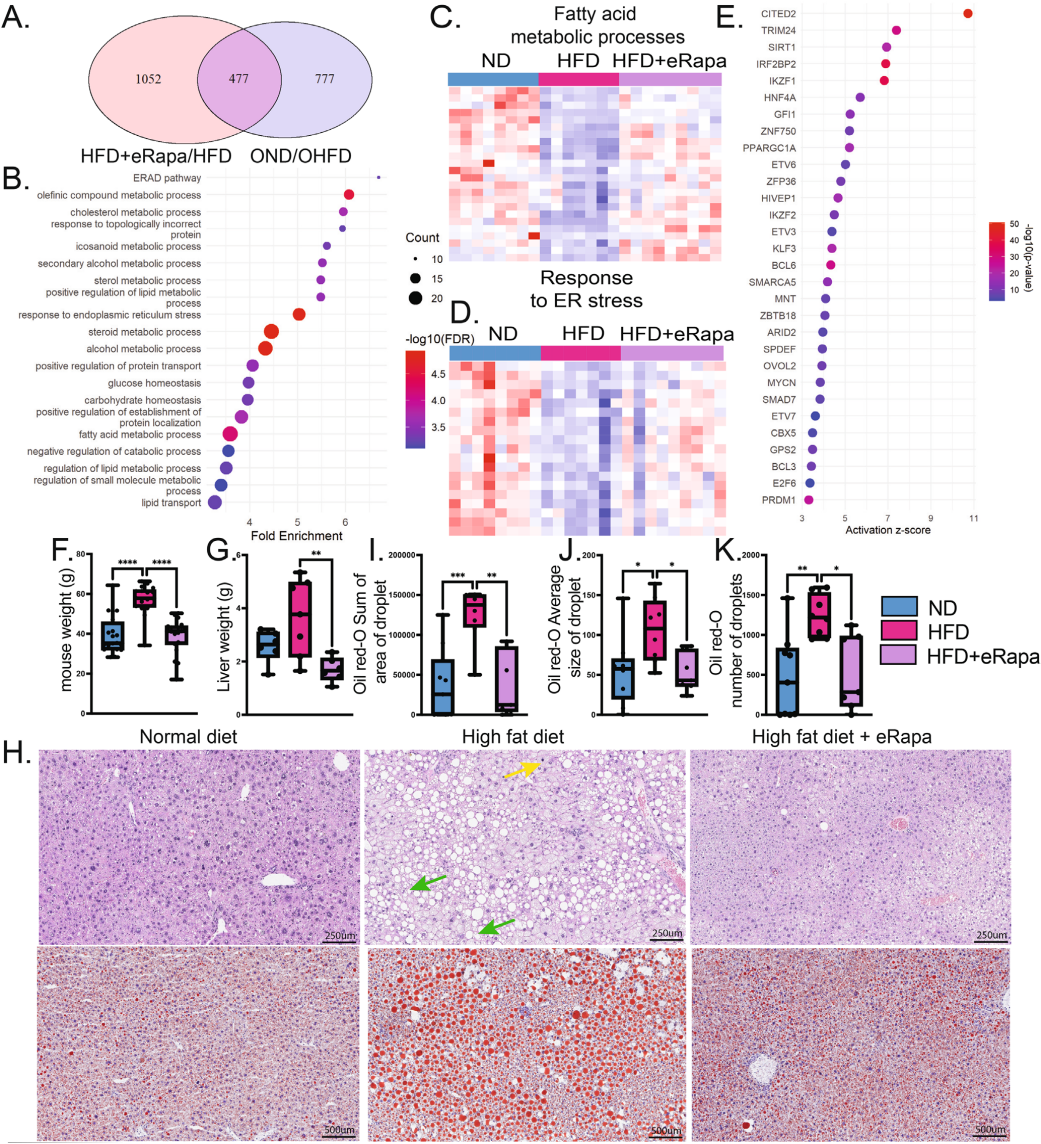
Rapamycin co‐treatment restores metabolic pathways prevents weight gain and steatosis in aged mice. (A) Venn diagram of overlapping genes downregulated by HFD versus ND and upregulated by eRapamycin; *p* = 3.49e–88, fold enrichment = 6.615. (B) KEGG enrichment analysis of the 477 overlapping genes. (C) Heatmap of genes involved in fatty acid metabolic processes. (D) Heatmap of genes associated with ER stress response. (E) Ingenuity Pathway Analysis identifying key transcriptional regulators of the 477genes. (F, G) Body weight and liver weight (g) of mice. (H) Representative liver histology: H&E staining (top row) and Oil Red O staining (bottom row). Yellow arrows indicate hepatocyte ballooning; green arrows indicate lipid droplets. (I–K) Quantification of Oil Red O staining: Total lipid area (I), average droplet size (J), and droplet number (K). Statistical analyses for weights and Oil Red O quantifications using one‐way ANOVA with post hoc Tukey's test. Significance is denoted as follows: *P* < 0.05 (*), *p* < 0.1 (**), *p* < 0.001 (***), *p* < 0.0001 (****).

Analysis of whole liver and purified hepatocytes identified a cluster of genes (Figures 1 and 2, Cluster 3) that was down regulated by HFD in both young and old, but especially in old. Of these 1254 genes down regulated by HFD, 477 were rescued by rapamycin treatment of old mice (*p* value: 3.49e‐88 and Fold Enrichment: 6.6) (Figure 4A). Analysis of the 477 genes downregulated by HFD + veh and upregulated by HFD + eRapa revealed enrichment in metabolic pathways, including cholesterol, icosanoid, sterol, and fatty acid metabolism, as well as protein folding and degradation processes such as ER‐associated protein degradation (ERAD) and the ER stress response (Figure 4B). Pathways conserved from this analysis compared to Cluster 3 in Figure 2 (i.e., downregulated by HFD and upregulated by HFD + eRapa) include ERAD pathway, Olefinic compound metabolic processes, response to ER stress, steroid metabolic process, alcoholic metabolic processes and fatty acid metabolic processes. Heatmaps for fatty acid metabolic processes and response to ER stress demonstrated that these pathways in HFD + eRapa‐treated mice closely resembled those in the ND + veh group (Figure 4C,D). Ingenuity Pathway Analysis (IPA) identified key transcriptional regulators of these genes, including *Trim24*, *Sirt1*, and *Hnf4a*, which play important roles in liver metabolic homeostasis and the modulation of inflammation (Figure 4E) (Tian et al. 2024; Van Dender et al. 2025; Jiang et al. 2015). Collectively, these results further show that rapamycin antagonizes additional HFD‐induced transcriptomic changes in livers of aged mice, indicating a protective effect against metabolic dysfunction in aging.

Mice on HFD + veh exhibited significant weight gain, with a median final weight of 57.8 g compared to 35.3 g in the ND + veh group. In contrast, mice on HFD + eRapa had a reduced median final weight of 41.3 g (Figure 4F). Similarly, liver weights were highest in the HFD + veh group (3.7 g) compared to ND + veh (2.6 g), while HFD + eRapa showed reduced liver weight to 1.7 g (Figure 4G). Histological analysis with H&E and Oil Red O staining showed extensive lipid accumulation (Figure 4H, green arrows) and hepatocyte ballooning (Figure 4H, yellow arrows) in the livers of HFD + veh mice, whereas the addition of rapamycin markedly reduced lipid deposition, restoring liver morphology closer to that of the ND + veh group (Figure 4H–K). These findings demonstrate that rapamycin can assuage the response to metabolic stress in old mice, as reflected in normalization of metabolic transcriptional profiles and prevention of inflammation‐associated signaling, a reduction of total body weight and suppression of features of steatotic liver disease and NASH.

## 3 Discussion

This study demonstrates that aging amplifies components of the hepatic response to HFD‐induced metabolic stress and that rapamycin treatment effectively mitigates these HFD‐induced transcriptional and pathological changes in aged liver. Whole liver and hepatocyte‐specific RNA‐sequencing revealed that aging exacerbates the upregulation of pro‐inflammatory/immune signaling pathways by HFD, while promoting the downregulation of genes involved in metabolism, cytochrome p450 and steroid hormone biosynthesis. Rapamycin treatment of aged HFD‐fed mice reversed many of these changes, including inflammatory transcriptional programs (e.g., *Stat1* and *Rela*) and suppression of metabolic gene expression. Together, these findings position aging as a key modifier of liver susceptibility to dietary metabolic stress and highlight rapamycin as a promising intervention to restore metabolic homeostasis and prevent diet‐associated disease progression in the setting of old age.

Aging is associated with increased vulnerability to metabolic stress, which contributes to disproportionate risk for MASLD and related health disparities in older populations. Older adults experience physiologic changes that impair metabolic flexibility, including reduced mitochondrial efficiency, decreased insulin sensitivity, and chronic low‐grade inflammation (Sheedfar et al. 2014; Palmer and Jensen 2022; Zhang et al. 2023). These age‐related alterations heighten susceptibility to lipid accumulation, hepatic steatosis, and systemic metabolic dysfunction in conjunction with poor diets or other metabolic stressors (Nunes‐Souza et al. 2016; Gan et al. 2011; Bertolotti et al. 2014; Estes et al. 2018; Delire et al. 2016). Moreover, aging is accompanied by changes in liver cell composition and phenotype, including increased immune cell infiltration and hepatocyte senescence, which may contribute to MASLD progression (Singh et al. 2008; Baiocchi et al. 2021; Liu et al. 2024; Kiourtis et al. 2024; Ogrodnik et al. 2017). Clinical evidence indicates that older individuals are more likely to experience severe steatosis, fibrosis, and adverse outcomes associated with MASLD, yet are often underrepresented in therapeutic trials (Sanyal et al. 2021; Kim et al. 2015; Alqahtani and Schattenberg 2021). Understanding how age alters the liver's response to dietary metabolic stress is critical for developing interventions that address the specific vulnerabilities of older individuals.

A limitation of our study is that within the geometric framework of nutrition (GFN), our diets are not merely “high fat” versus “control,” but represent a pronounced rebalancing of macronutrients (Simpson et al. 2017). Most notably a ~20‐fold shift in the carbohydrate: fat ratio (control diet ~7 to HFD ~0.33). Such changes in nutrient balance can alter food intake regulation and metabolic set points, which could influence hepatic and transcriptomic outcomes beyond fat content alone. Thus, in a more general sense, our study demonstrating the interactions of age and HFD is just one specific example of how age and diet composition can interact with each other. With the current experimental design, we cannot formally identify which aspect(s) of the changing diet is interacting with age, the change in fat content or the change in carbohydrate: fat ratio. However, in the case of the rapamycin study, our primary conclusions derive from pair‐wise comparisons within an identical macronutrient context: HFD + veh versus HFD + eRapa, thereby eliminating the confounding effects of diet composition and allowing us to attribute group differences to rapamycin treatment rather than to nutrient geometry. An additional limitation is that albumin content was assessed in liver homogenates as a surrogate index of hepatocyte synthetic function, because serum was not collected at the time of the study. Unlike serum albumin, which is widely used as a sensitive marker of hepatic protein synthesis and global liver function, the intrahepatic albumin pool is relatively buffered and changes more slowly in response to metabolic or inflammatory stress. Also, measurement of liver homogenate albumin can be influenced by hepatocyte number, total protein content, and albumin trafficking as well as synthesis. Albeit with these caveats, we did not detect any significant differences in hepatic albumin content between old mice maintained on ND, HFD + veh, or HFD + eRapa. This provides some reassurance that the dietary and pharmacological interventions used here do not cause gross impairment of hepatocyte function in aged animals.

Liu et al. (2014) reported that chronic rapamycin treatment increased fat mass and body mass on HFD. In our study, rapamycin reduced body and liver mass of HFD‐fed mice. As potential reconciliation of this discrepancy, we note that Liu et al. (2014) used 14 ppm eRapa for several months in young mice, while we used 42 ppm eRapa beginning at 4 months of age until euthanasia at 20 months. Conceivably, the difference in dose of eRapa and age of the mice is responsible for the observed difference in body weight change. Notably, like our study, prior studies have reported rapamycin‐induced weight loss (Leontieva et al. 2014a; Schindler et al. 2014; den Hartigh et al. 2018). Importantly, weight loss in these studies occurred despite equal food intake or even hyperphagy, suggesting that rapamycin‐induced weight loss is not due to decreased food intake and caloric restriction (Leontieva et al. 2014a; Schindler et al. 2014; den Hartigh et al. 2018). Although in our study we did not measure food consumption, chow was replaced weekly, food consumption appeared comparable between cohorts, and visual inspection at necropsy consistently showed diet coloring throughout the gastrointestinal tract across cohorts. Consequently, weight loss due to decreased food consumption seems unlikely. Even so, some of the observed transcriptomic changes associated with rapamycin might still be a secondary consequence of weight loss rather than a direct pharmacologic effect of rapamycin in the liver. However, we observed marked effects of rapamycin on biochemical and transcriptomic markers of mTOR activity in liver, making it seem unlikely that all effects of rapamycin are secondary to weight loss. In sum, our observation of rapamycin‐induced body weight loss is in line with several, but not all, previous studies and is unlikely to be due to decreased food intake.

HFD has been previously noted to recapitulate multi‐system aging phenotypes, including hepatic dysfunction (Newman et al. 2015). Indeed, previous studies have investigated the effects of candidate longevity/healthy aging interventions on effects of diet, including HFD. Resveratrol given to mice on high‐calorie/HFD improves survival and metabolic health while remodeling hepatic gene expression and protecting against steatosis, establishing an early precedent for liver transcriptomic effects under HFD plus a candidate longevity compound (Baur et al. 2006). Chronic rapamycin feeding produces major pathway‐level reprogramming of the liver transcriptome that is enhanced when combined with dietary restriction, demonstrating potent diet–drug interactions at the hepatic omics level (Fok, Chen, et al. 2014; Fok, Bokov, et al. 2014). Extending from transcript to protein, a proteomics atlas across 40 diet formulations showed that diet is the dominant driver of hepatic proteome state, with rapamycin, resveratrol, and metformin modifying diet‐evoked proteomic programs, again underscoring that longevity/healthy aging drugs reshape liver responses to nutrient environment rather than acting in isolation. However, few studies have investigated the effects of candidate longevity/healthy aging interventions on dietary stress in aged mice, as we have done here (Leontieva et al. 2014a, 2014b; Blagosklonny 2008).

Rapamycin has emerged as a promising intervention capable of simultaneously targeting age‐related decline and maladaptive responses to metabolic stress. Through inhibition of mTORC1, rapamycin suppresses anabolic pathways and can promote a shift toward oxidative metabolism and mitochondrial efficiency (Zhang, Xiao, et al. 2025). It enhances fatty acid oxidation and activates transcriptional regulators like *Pparα* and *Sirt1*, supporting improved lipid metabolism and stress resilience. In preclinical models of aging and metabolic disease, rapamycin has been shown to restore metabolic gene expression, reduce adiposity, and alleviate hepatic steatosis (Chang et al. 2009; Bitto et al. 2021; Leontieva et al. 2014a, 2014b). While the benefits of rapamycin have been characterized in either metabolic disease or aging models individually, they have not been thoroughly explored in the context of their intersection, where aging amplifies vulnerability to metabolic stress. Our findings position rapamycin as a therapeutic candidate capable of restoring metabolic resilience in the aged liver, offering a strategy to counteract the compounding effects of age and dietary stress on metabolic health.

Among the pathways most strongly affected by age and HFD in both whole liver and isolated hepatocytes was the degradation of branch chain amino acids (BCAA): valine/leucine/isoleucine. The aged transcriptome was enriched for expression of genes involved in BCAA degradation and this was offset by HFD. Evidence indicates that reducing BCAA burden, either by restricting dietary BCAAs or by activating BCAA catabolism with branched‐chain ketoacid dehydrogenase kinase (BCKDK) inhibitors, ameliorates steatosis and diet‐induced obesity in mice (Cummings et al. 2018; Fontana et al. 2016; Zhang, Xu, et al. 2025; Bollinger et al. 2022; Acevedo et al. 2024; Roth Flach et al. 2023). In humans with MASLD/MASH, hepatic BCKDK expression and circulating BCCAs are elevated and associate with disease severity, with SREBP1 acting as a transcriptional activator of BCKDK (Grenier‐Larouche et al. 2022). Because rapamycin suppresses mTORC1–SREBP1 lipogenesis (Crewe et al. 2019), one can propose an extended model in which rapamycin lowers BCKDK, activates branched‐chain alpha‐keto acid dehydrogenase complex (BCKDH), and enhances BCAA degradation, thereby reducing lipogenic drive (Crewe et al. 2019; Porstmann et al. 2008). This convergence across diet mTORC1 inhibition points to mTORC1 → SREBP1 → BCKDK/BCKDH as a potential mechanistic axis linking rapamycin to improved hepatic metabolism.

Our results suggest that rapamycin can have dual benefit through its ability to promote healthy tissue function and reduce cancer risk. Steatosis is a risk factor for MASLD progression and liver cancer development. Histological analysis confirmed that rapamycin reduced liver steatosis and inflammation associated with HFD feeding in aged animals. Interestingly, rapamycin treatment of HFD mice also resulted in a marked reduction in liver tumorigenic index scores (Figure 3M), a marker of liver cancer risk (Wang et al. 2019). Our findings align with previous work on rapamycin increasing lifespan and delaying spontaneous tumor incidence in mice (Harrison et al. 2009; Blagosklonny 2008; D'adda Di Fagagna 2008). Thus, rapamycin may serve as a gerotherapeutic that simultaneously promotes metabolic health and cancer resistance in aging.

This study identifies aging as a key enhancer of liver vulnerability to dietary stress and highlights rapamycin as a potent modulator of this response. Aging exacerbates liver transcriptional responses of metabolic dysfunction, inflammation, and tumor risk when exposed to HFD. Rapamycin restores metabolic gene expression and suppresses inflammatory programs in aged hepatocytes of HFD fed mice. This intervention reduces liver steatosis, body weight, and tumorigenic risk scores. Findings support broader application of gerotherapeutics in suppressing combined age‐ and diet‐related metabolic dysfunction and cancer risk. These results provide rationale for use of gerotherapeutics as a disease‐modifying strategy in diet‐induced metabolic disease in older people.

## 4 Experimental Procedures

### 4.1 Animal Usage

All animal procedures were approved by the Institutional Animal Care and Use Committee (IACUC) of Sanford Burnham Prebys Medical Discovery Institute. Animal experiments were performed at Sanford Burnham Prebys Medical Discovery Institute Animal Facility in compliance with the IACUC guidelines. The studies performed within this manuscript were performed with all relevant ethical regulations regarding animal research. Young and old C57BL/6 animals were obtained from the NIA aging colony housed at Charles Rivers or breed within the institute or were purchased from Charles Rivers. Animals were housed 5 mice per cage and maintained under controlled temperature (22.5°C) and illumination (12 h dark/light cycle) conditions. High‐fat diet utilized within this study was obtained from Test Diet 58Y1 consisting of 60% calories from fat or “normal diet” 58Y2, 10% calories from fat. Microencapsulated rapamycin (42 ppm) and eudragit control were purchased from Emtora (formerly Rapamycin Holdings) and compounded into the appropriate diet by NewCO Distributors. Treatment is similar as described previously (Wilkinson et al. 2012; Cole et al. 2017; Wang et al. 2017). Mice were maintained on diet ad libitum until the time of euthanasia; diet was replaced weekly.

### 4.2 Hepatocyte Isolation

Following a protocol adapted from Charni‐Natan and Goldstein 2020, mice were euthanized via CO_2_ asphyxiation, and once immobilized, the abdominal cavity was opened to expose the inferior vena cava. A 24G × ¾″ catheter (Surflo, SR‐0X2419CA) was inserted into the vein, and the liver was perfused with 50 mL of pre‐warmed (42°C) HBSS (Gibco, Ref #14175–095) at a flow rate of 6 mL/min, followed by 40 mL of DMEM (Gibco, Ref #10313–021) containing Collagenase Type IV (Gibco, Ref #17104–019), also pre‐warmed to 42°C. After digestion, livers were excised and gently dissociated in ice‐cold DMEM supplemented with 2% Bovine Serum Albumin (BioWorld, CAS: 9048‐46‐8) and kept on ice. Cells were washed by centrifugation at 50 × g for 2 min at 4°C, with media changes repeated five times. The final cell suspension was resuspended in a gradient solution consisting of 40% Percoll (Cytiva, Ref #17089102), 10% 10× HBSS (Gibco, Ref #14065–056), and 60% DMEM, and centrifuged at 100 × g for 7 min at 4°C. Viable hepatocytes from the pellet were collected, washed once in DMEM at 50 × g, and counted using a hemocytometer with trypan blue exclusion for viability assessment.

### 4.3 Histology, Immunofluorescence and Staining

Picro‐Sirius Red staining was performed on 5 μm formalin‐fixed paraffin‐embedded (FFPE) liver sections following the protocol detailed by Emory University Microscopy in Medicine (see: link). For immunofluorescence, formalin‐fixed paraffin‐embedded sections were de‐paraffinized with 2× 5‐min washes in xylene followed by a 5‐min incubation in 100%, 90%, and 70% ethanol. Antigen retrieval using buffer (Tris, EDTA) was conducted using a steamer for 45 min. Primary antibodies: CD3 (Abcam, ab16669), CD45 (R&D Systems, clone 30‐F11) were incubated at 4°C overnight. Secondary antibodies Anti‐Mouse Alexa Fluor 594 (Invitrogen, A11032) and Anti‐Rabbit Alexa Fluor 488 (Invitrogen, A11008) were incubated at room temperature for 1 h in the dark followed by 15 min of DAPI and cover slip with Fluoromount‐G (Southern Biotech, 0100–01) and images using Nikon Eclipse Ti2 microscope.

### 4.4 Western Blotting

Homogenized tissue or cell suspensions were quantified using the Bradford assay (Pierce, Ref #1863028) on a SpectraMax 190 plate reader. SDS‐PAGE gels were freshly cast and run following the protocol described by Lynch et al. (PMID: 27791595) using the Bio‐Rad Mini‐PROTEAN Tetra System. Proteins were transferred to PVDF membranes (Immobilon‐PSQ, Millipore ISEQ00010) at 100 V for 70 min. Membranes were stained with Ponceau S (Sigma‐Aldrich, P7170‐1 L) prior to blocking with 5% skim milk (BD Difco, Ref #232100) in TBST for 1 h at room temperature. Primary antibodies: Phospho‐S240/244‐S6 (Cell Signaling Technologies, 5364), S6 Ribosomal protein (Cell Signaling Technologies, 2312), Stat1 (Cell Signaling Technologies, 9172) were incubated in 5% BSA (BioWorld, Ref #9048‐46‐8) overnight at 4°C with rocking. Secondary antibodies used were Goat anti‐Mouse IgG‐HRP (Thermo Fisher Scientific, Cat# 31446, RRID:AB_228318) and Goat anti‐Rabbit IgG‐HRP (Millipore, Cat# AP307P, RRID:AB_92641). Membranes were imaged for Ponceau S and HRP signal using a Bio‐Rad ChemiDoc Touch system, and images were processed and analyzed with Image Lab software. Antibodies used: total Stat1 (Cell Signaling Technologies 9172S), S6 Ribosomal protein (Cell Signaling Technologies 2317), phospho‐S6 Ribosomal protein (Cell Signaling Technologies 5364).

### 4.5 ELISA

Equal amounts of whole mouse liver homogenate were generated and examined (normalized to total protein quantity) using Mouse Albumin ELISA Kit RayBiotech (ELM‐Albumin‐1) as per company recommendations. Albumin in 96 plates was measured by 450 nm in Plate Reader (Epoch).

### 4.6 RNAseq Analysis

Raw fastq files were aligned to mm10 (Gencode vM23), using STAR (Dobin et al. 2013) 2‐pass pipeline. Aligned reads were filtered, sorted, and indexed by SAMtools V1.1.0 (Danecek et al. 2021). Genome tracks (bigWig files) were obtained by Deeptools V3.3.2 (Ramirez et al. 2016). Raw read counts were obtained by HTSeq V0.11.2 for differential analysis. Differentially expressed genes were obtained by DESeq2 V1.26.0 (Love et al. 2014) CuffLinks V2.2.1Trapnell (Trapnell et al. 2010) was used to compute FPKM values.

### 4.7 Clustering and Visualization of Differentially Expressed Genes

Differentially expressed genes (DEGs) were clustered based on their normalized FPKM expression values using hierarchical clustering with Pearson correlation distance and average linkage. Genes with missing or zero expression were removed, and expression values were scaled by row. Clusters were defined by cutting the dendrogram into groups for each gene cluster; normalized expression profiles were visualized by min‐max scaling each gene's expression across samples and plotting both individual gene trajectories and the average expression profile. These plots were used to assess shared expression dynamics across experimental groups.

### 4.8 Principal Component Analysis

PCA was performed on log_2_‐transformed FPKM values (with a pseudocount of 1) after transposing the matrix to set samples as rows. Genes with zero variance were excluded. PCA was conducted using the prcomp function in R, and the first three principal components were visualized using an interactive 3D scatter plot generated with the plotly package. Samples were colored by group to assess separation based on expression profiles.

### 4.9 Gene Set Overlap and Functional Enrichment Analysis

Overlapping up‐ and downregulated genes from two differential expression comparisons were identified and visualized using Venn diagrams (VennDiagram), with overlap significance calculated via hypergeometric testing and high‐precision *p* values (Rmpfr). Fold enrichment was computed using a mouse genome background of 32,179 genes.

### 4.10 GO and KEGG Enrichment Analyses

GO and KEGG enrichment analyses were conducted with clusterProfiler (*q* < 0.05, BH correction), and top terms were visualized using dot plots showing fold enrichment and –log_10_ adjusted *p* values. For selected GO terms, *Z*‐score–normalized expression values were used to generate heatmaps of associated genes across experimental groups.

### 4.11 Ingenuity Pathway Analysis (IPA)

Upstream regulator analysis was performed using Ingenuity Pathway Analysis (IPA) and filtered to include only transcription factors. Transcription factors with significant overlap (*p* < 0.05) and non‐zero activation z‐scores were retained. The top 30 predicted activators and inhibitors were ranked by activation z‐score and visualized as dot plots, with color indicating –log_10_ *p* value. Positive *z*‐scores reflect predicted activation, while negative *z*‐scores indicate predicted inhibition.

### 4.12 Correlation Analysis of Gene Expression Changes

To assess how Rapamycin modulates gene expression changes induced by a high‐fat diet (HFD), log_2_ fold changes (log_2_FC) were calculated for ND versus HFD and HFD versus Rapamycin using normalized FPKM values. The analysis included genes that were differentially expressed in the HFD condition relative to ND. Log_2_FC values were computed using group means and a pseudocount (ε = 1e–3) to avoid division by zero. A scatter plot was generated to compare the two contrasts, and Pearson correlation was used to quantify their relationship.

### 4.13 Use of Artificial Intelligence Technology

ChatGPT was used for grammatical and formatting purposes with human oversight. The ideas and development of the manuscript along with results and conclusions were generated and reviewed by authors on the manuscript prior and post writing.

## Author Contributions

Aaron Havas and Adarsh Rajesh contributed equally for the development, actualization and execution of experiments along with data analysis and writing of the manuscript. Xue Lei: contributed computational analysis and manuscript writing. Jessica Proulx, Adam Field, Andrew Davis, Marcos Garcia Teneche, and Armin Gandhi: contributed experimental execution and manuscript writing. Karl N. Miller: contributed experimental design and manuscript writing. Jin Lee and Gen‐Sheng Feng: contributed experimental execution and manuscript writing for the revisions of the manuscript. Peter D. Adams: contributed experimental design, data analysis, data interpretation, and manuscript writing and revisions.

## Funding

This work was supported by National Institute of Aging (P01 AG031862, P01 AG073084, and U54 AG079758), American Society of Hematology, National Cancer Institute (T32 CA211036) and Cancer Center Support Grant to Sanford Burnham Prebys (P30 CA030199‐44), for support of Core Facilities (Animals, Histology, Flow Cytometry and Genomics).

## Conflicts of Interest

The authors declare no conflicts of interest.

## Supporting information


**Figure S1:** Aging exacerbates high‐fat diet (HFD)‐induced metabolic stress responses. (A) Immunofluorescence of young (5‐month‐old) and aged (22‐month‐old) mouse liver after 9 weeks of HFD examining Cd45 (green) and Cd3 (red) expression within large tertiary lymphoid‐like structures and immune infiltration observed in old mouse with HFD.
**Figure S2:** CYBRSORTx representation of immune transcripts in whole mouse liver RNAseq young (5‐month‐old) and aged (22‐month‐old) mice after 9 weeks of HFD or control diet. Statistical analysis was performed using one‐way ANOVA with post hoc Tukey's test; *p* < 0.05 is indicated by an asterisk (*), *p* < 0.05 (*), *p* < 0.1 (**), *p* < 0.001 (***), *p* < 0.0001 (****).
**Figure S3:** HFD+eRapa abrogates gene expression associated with aging, reduces mTor signaling and SASP in old mouse hepatocytes. (A) ELISA‐measured albumin concentration (450 nm absorbance) in whole liver homogenates, with equal total protein loaded per sample. (B–E) Heatmap and quantitated score of mTor target gene expression of (B, C) GO, and (D, E) KEGG. (F, G) GO BCAA catabolism. (H, I) KEGG BCAA degradation in isolated hepatocytes. (J) Scatter plot comparing gene expression changes induced by age (young ND vs. old ND) to all gene expression changes with eRapamycin (eRapa + HFD vs. HFD + veh) hepatocytes. (K, L) Heatmap of SASP gene expression. Statistical analysis used to compare mouse cohorts was one‐way ANOVA with post hoc Tukey's test.

## Data Availability

All data generated for this manuscript is available publicly from the Gene Expression Omnibus (GEO) repository. The whole liver young versus old ± HFD (Figure 1) RNAseq accession number is GSE313644. The isolated hepatocyte young vs. old ± HFD RNAseq (Figure 2) accession number is GSE243906 and the old HFD + Rapamycin RNAseq (Figures 3 and 4) accession number is GSE316732.
