# Supplementary material for: Rapamycin Reverses the Hepatic Response to Diet‐Induced Metabolic Stress That Is Amplified by Aging
Source: Aging Cell. 2026 Feb 7;25(2):e70395. doi: 10.1111/acel.70395 (PMC12882742; doi:10.1111/acel.70395)
Supplement: Supplementary file 1 — Figure S1: Aging exacerbates high‐fat diet (HFD)‐induced metabolic stress responses. (A) Immunofluorescence of young (5‐month‐old) and aged (22‐month‐old) mouse liver after 9 weeks of HFD examining Cd45 (green) and Cd3 (red) expression within large tertiary lymphoid‐like structures and immune infiltration observed in old mouse with HFD. Figure S2: CYBRSORTx representation of immune transcripts in whole mouse liver RNAseq young (5‐month‐old) and aged (22‐month‐old) mice after 9 weeks of HFD or control diet. Statistical analysis was performed using one‐way ANOVA with post hoc Tukey's test; p < 0.05 is indicated by an asterisk (*), p < 0.05 (*), p < 0.1 (**), p < 0.001 (***), p < 0.0001 (****). Figure S3: HFD+eRapa abrogates gene expression associated with aging, reduces mTor signaling and SASP in old mouse hepatocytes. (A) ELISA‐measured albumin concentration (450 nm absorbance) in whole liver homogenates, with equal total protein loaded per sample. (B–E) Heatmap and quantitated score of mTor target gene expression of (B, C) GO, and (D, E) KEGG. (F, G) GO BCAA catabolism. (H, I) KEGG BCAA degradation in isolated hepatocytes. (J) Scatter plot comparing gene expression changes induced by age (young ND vs. old ND) to all gene expression changes with eRapamycin (eRapa + HFD vs. HFD + veh) hepatocytes. (K, L) Heatmap of SASP gene expression. Statistical analysis used to compare mouse cohorts was one‐way ANOVA with post hoc Tukey's test. [file ACEL-25-e70395-s001.pdf]

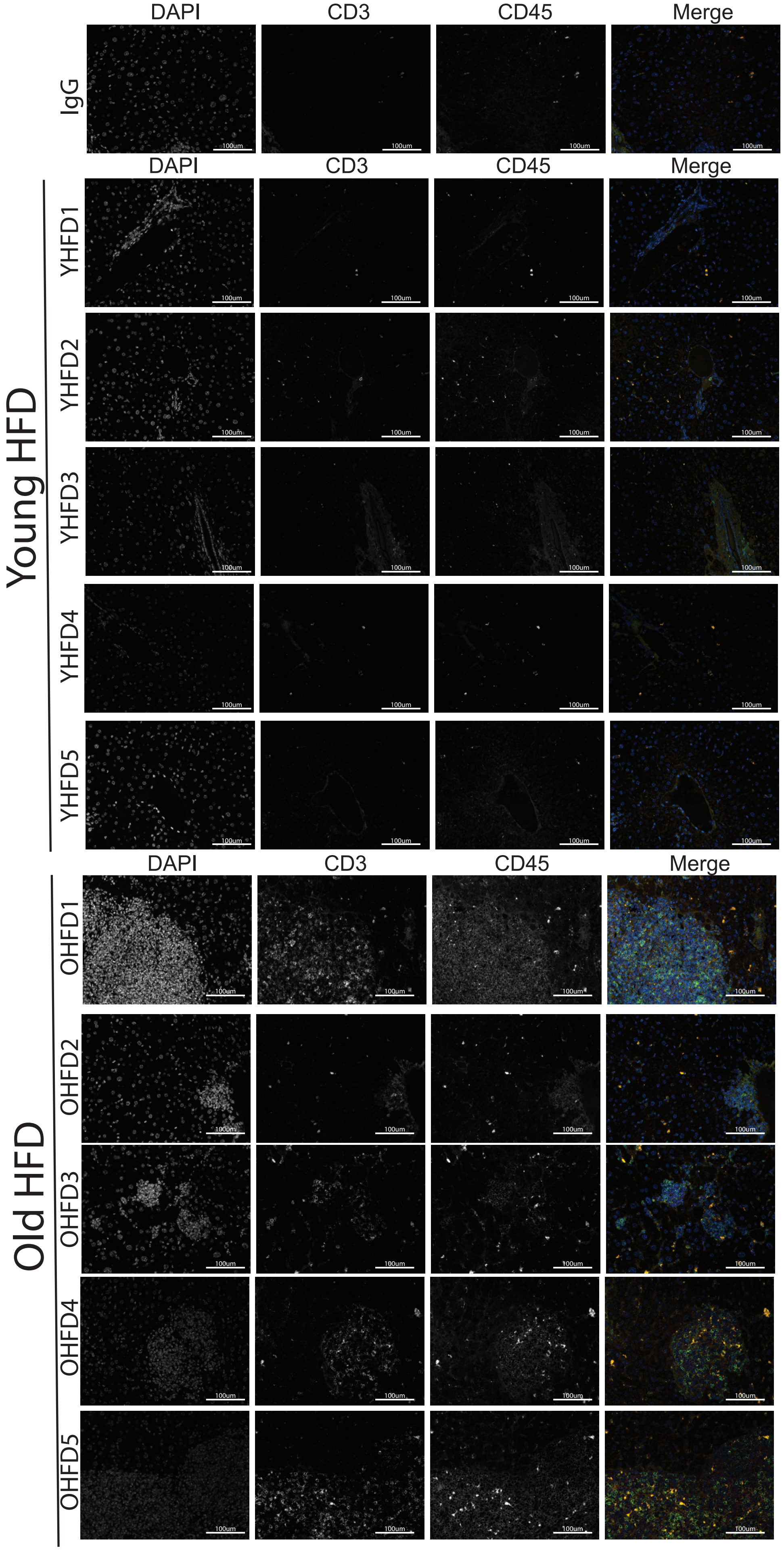

Sup Fig 1. Aging exacerbates high-fat diet (HFD)-induced metabolic stress responses.
(A) Immunoflourescence of young (5-month-old) and aged (22-month-old) mouse liver after 9 weeks of HFD examining Cd45 (green) and Cd3 (red) expression within large tertiary lymphoid-like structures and immune infiltration observed in old mouse with HFD.

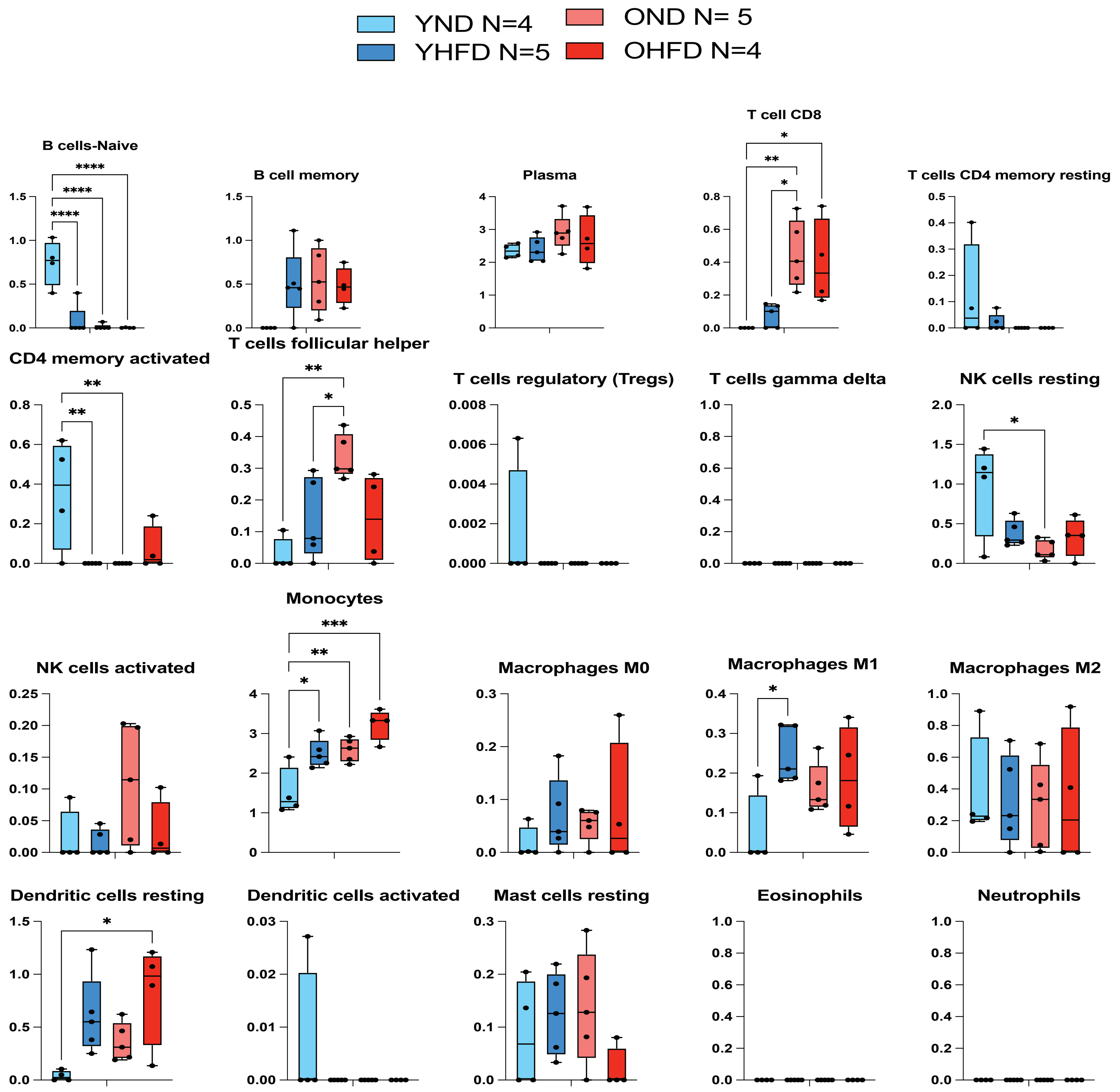

Sup Fig 2 CYBRSORTx representation of immune transcripts in whole mouse liver RNAseq young (5-month-old) and aged (22-month-old) mice after 9 weeks of HFD or control diet. Statistical analysis was performed using one-way ANOVA with post hoc Tukey's test; $p < 0.05$ is indicated by an asterisk (*), $p < 0.05$ (*), $p < 0.1$ (**), $p < 0.001$ (***), $p < 0.0001$ (****).

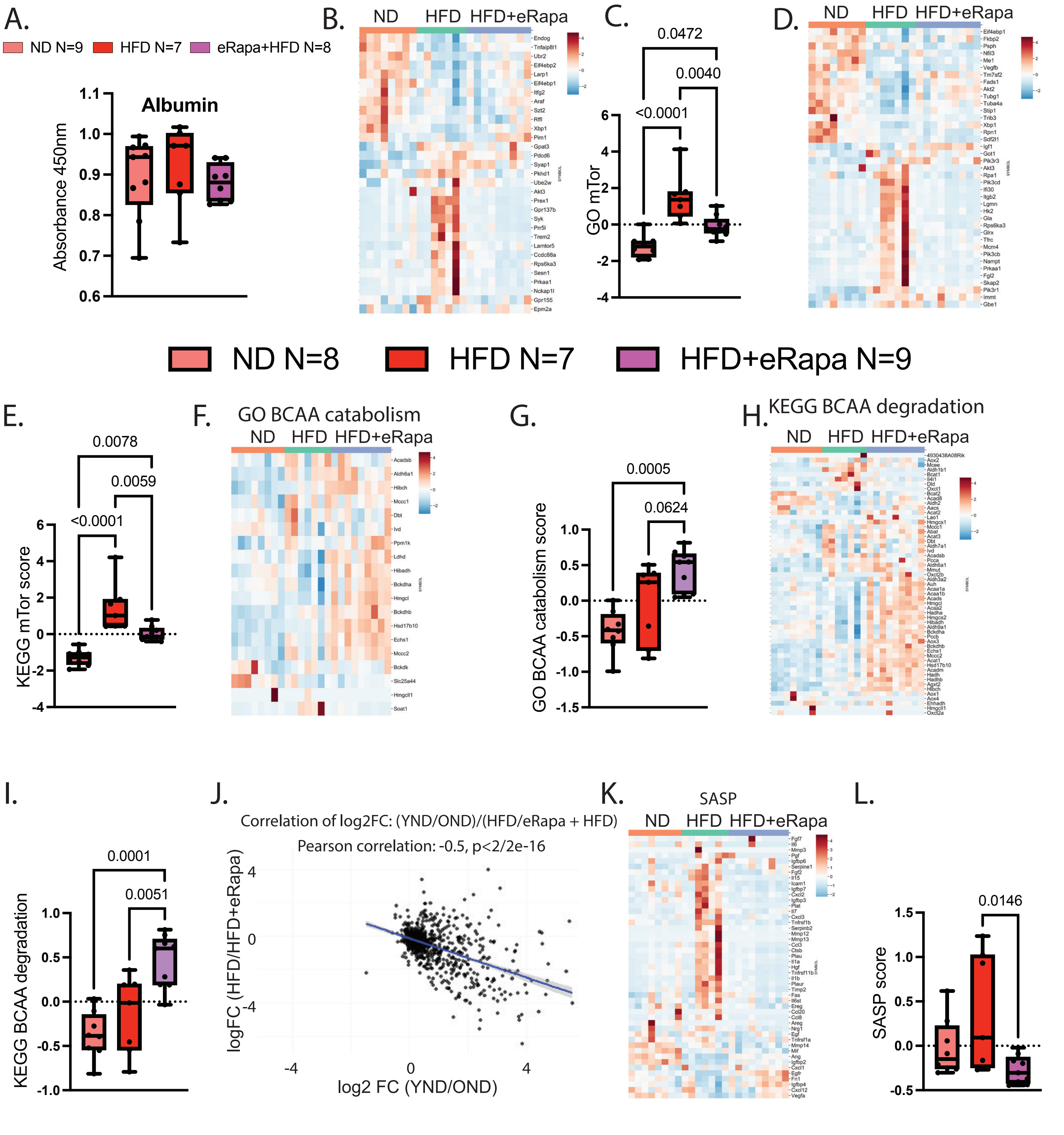

Sup Fig 3 HFD+eRapa abrogates gene expression associated with aging, reduces mTor signaling and SASP in old mouse hepatocytes. (A) ELISA-measured albumin concentration (450 nm absorbance) in whole liver homogenates, with equal total protein loaded per sample. (B-E) Heatmap and quantitated score of mTor target gene expression of (B-C) GO, and (D-E) KEGG . (F-G) GO BCAA catabolism. (H-I) KEGG BCAA degridation in isolated hepatocytes. (J) Scatter plot comparing gene expression changes induced by age (young ND vs old ND) to all gene expression changes with eRapamycin (eRapa+HFD vs HFD+veh) hepatocytes. (K-L) Heatmap of SASP gene expression. Statistical analysis used to compare mouse cohorts was one-way ANOVA with post hoc Tukey's test.
